# Public Involvement in Global Genomics Research: A Scoping Review

**DOI:** 10.3389/fpubh.2019.00079

**Published:** 2019-04-09

**Authors:** Jack S. Nunn, Jane Tiller, Peter Fransquet, Paul Lacaze

**Affiliations:** ^1^School of Psychology and Public Health, La Trobe University, Melbourne, VIC, Australia; ^2^Public Health Genomics, School of Public Health and Preventive Medicine, Monash University, Melbourne, VIC, Australia

**Keywords:** genomics research, public health, public involvement, scoping review, co-design and co-production, patient and public involvement and engagement, patient participation [MeSH term], public health genomics

## Abstract

Public involvement in research occurs when the public, patients, or research participants are actively contributing to the research process. Public involvement has been acknowledged as a key priority for prominent human genomics research initiatives in many different countries. However, to date, there has been no detailed analysis or review of the features, methods, and impacts of public involvement occurring in human genomics research projects worldwide. Here, we review the reported public involvement in 96 human genomics projects (initiatives), based on a database of initiatives hosted by the Global Alliance for Genomics and Health, according to information reported on public domain websites. To conduct the scoping review, we applied a structured categorization of criteria to all information extracted from the search. We found that only a third of all initiatives reported public involvement in any capacity (32/96, 33%). In those reporting public involvement, we found considerable variation in both the methods and tasks of involvement. Some noteworthy initiatives reported diverse and comprehensive ways of involving the public, occurring through different stages of the research project cycle. Three notable initiatives reported a total of eight distinct impacts as a result of involving people. Our findings suggest there would be intrinsic value in having more public involvement occur in human genomics research worldwide. We also suggest that more systematic ways of reporting and evaluating involvement would be highly beneficial, to help develop best practices.

## Introduction

In human genomics, there is a growing need to increase involvement of the public in research and policy development. This has been identified as a crucial aspect of responsible research practice (1, 2). The concept of “public involvement” in research is defined as research that is carried out “with” people rather than “on” them (3). Public involvement can also be defined as when the public, patients or research participants actively contribute to the research or policy development process (4).

The number of people involved in genomics research is predicted to grow substantially in coming years (5, 6). By 2025, it is estimated that nearly 2 billion people worldwide will have had their DNA sequenced, creating a global imperative for responsible and effective public involvement in research (7). Many high-profile genomics research initiatives have already made public statements about the importance of involving people, with some governments positioning public involvement as a democratic right (8–10). For example, in the report “Generation Genome,” the UK's Chief Medical Officer suggested that shaping the future of genomic research requires the “active involvement of many stakeholders including patients, health professionals, researchers, policymakers, and wider society,” with a “key role for public engagement and involvement” (10).

The benefits of involving the public in research are wide-ranging. They include improving trust and public influence over research (1, 7, 8); ensuring that research is conducted in an ethical, accessible and transparent manner; and ensuring that research reflects the balance and diversity of priorities within populations (11, 12). However, with the growing interest and importance of large-scale human genomics initiatives worldwide, there has been limited research into how the public are currently being involved. There has also been no structured assessment of the resulting impacts and benefits, including genomics initiatives that have involved the public.

While involving the public in other types of health and medical research has been the subject of previous systematic reviews (13–15), comparable reviews have not been published in human genomics. Many of the existing reviews on other areas of medical research conclude that reports of involvement activities are inconsistent or under-reported (15–19) and that the precise ways in which people are involved in medical research are not well-reported, including any impacts from involving people (7, 14, 16).

Our review provides a summary of reported public involvement in 96 global human genomics projects, listed on a database managed by the Global Alliance for Genomics and Health (GA4GH), a recently formed international organization seeking to enable responsible genomics data sharing within a human rights framework (20). The list provides a representation of the current landscape of human genomics research worldwide, and a snapshot of contemporary practice with regards to public involvement in human genomics research.

This scoping review provides a new perspective by exploring how these genomics initiatives have conducted and reported public involvement to date, including any impacts, facilitators and barriers of involvement. The intention is that resulting data will help inform future directions for integrating public involvement into genomics research and policy development, and inform the development of ways of routinely reporting and evaluating any involvement.

## Methods

### Source

**Figure 1:**
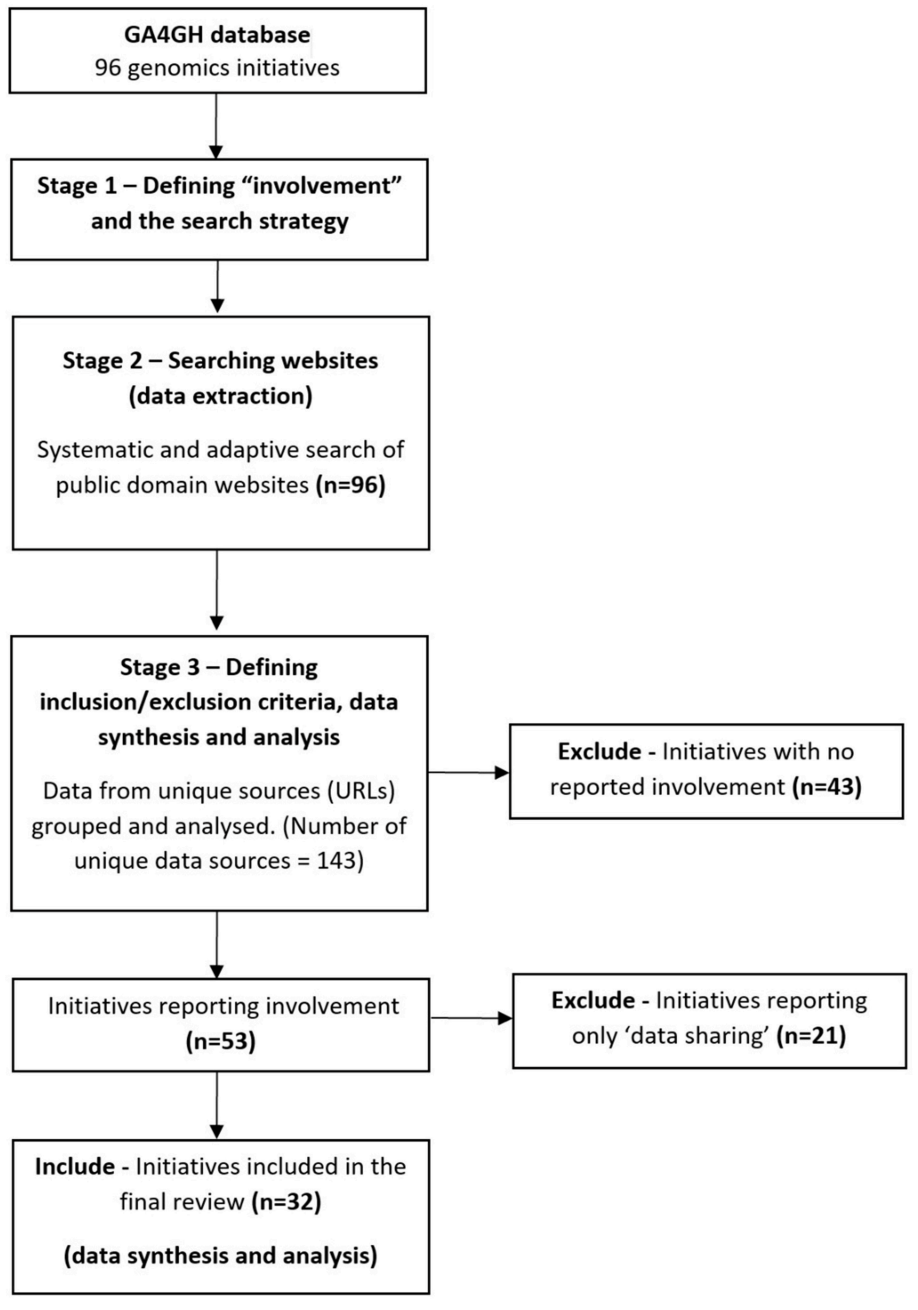
Scoping review study overview and results summary.

Using a list of human genomics research projects (referred to as “initiatives”) from a database hosted by the GA4GH (see Table S1), we systematically searched public domain websites for information reported on involving the public in research. The database was curated by GA4GH staff, last verified August 2016, and contains information about the type of the genomics research initiative (i.e., consortium, data-sharing initiative, organization(s), repository or research project), the type of data gathered (i.e., whole-genome or whole-exome sequencing), the geographical scope of the initiative, number of participants (cohort size), relevant disease areas, and the public domain URL of the website for the organization or initiative (as some “initiatives” involve a number of organizations). The scoping review methodology can be summarized in three stages (see Figure 1).

### Stage 1—Defining “Involvement” and the Search Strategy

We developed a criteria to define “involvement” and refine our search terms, informed by the International Association for Public Participation's participation spectrum and other studies (4, 8, 21, 22). This included reports of “consultation,” “involvement,” “collaboration,” and “empowerment” (23). Involving people in genomics research was defined as the “active involvement” in shaping and guiding research, rather than only providing data (17, 23, 24). We defined specific tasks related to involvement at different stages of the research cycle (3), such as the sharing of views to influence research, or co-creating the research (19, 25, 26). “Consequential” involvement is when involvement contributes to the research process, as distinct from involvement which is ignored or not incorporated (27–29). We could not always determine whether involvement was consequential based on the available information, so an assumption was made that all methods reported were “consequential.”

### Stage 2—Searching Websites (Data Extraction)

Public domain websites of all the initiatives in the GA4GH database were searched for reports of involvement and associated impacts. The date range for website searching and data extraction was 16th August to 28th November 2017. The exact text from the URLs where data was extracted from was collected to allow reanalysis, with all relevant URLs archived using an online archive service to preserve the content and the date of extraction (30).

After a manual search of each domain, search engine operators were entered into the Google “site search” function in order to systematically scan the text of each public domain website for relevant phrases, including all grammatical variations of the words used (for example, deriving “involvement,” “involves,” “involved,” and “involving” from the root word “involve”). Grammatical variations of specific phrases (denoted by inverted commas) were generated using tables to systematically create a series of search strings for each domain. For example, this search string returned 4 results:

*site:**www.ukbiobank.ac.uk/* “public involvement” OR “involves public” OR “public involved” OR “involving the public” OR “involve public”

Reports of involvement were assessed by a member of the research team (JN), then independently assessed by an additional member of the research team, with a random sample assessed by a third investigator (PL). Any disagreements between the team on the data included was discussed until a consensus was reached. Informed by previous reviews, the search terms for the concept of involvement were; “engagement,” “involvement,” and “partnership” (21, 31–34). The search terms to describe the people involved were; “citizen(s),” “community,” “consumer(s),” “lay,” “patient(s),” “public,” “stakeholder(s),” and “user(s).”

In addition to using a standard list of terms, adaptive (context dependent) search terms were sometimes required when searching domains where terms were specific to the region or initiative. Adaptive search terms were; “advocate(s),” “carer(s),” “civil society,” “client(s) (35),” “customer(s) (35),” “group(s),” “participant(s),” “payer(s),” “population(s) (29),” “PPI” (an acronym commonly used in the UK which stands for “patient and public involvement”), “residents” (geographical grouping) (36), “representative(s),” “taxpayer(s),” and “volunteer(s).” For more details on search method, see Systematic search method.

### Stage 3—Defining Inclusion/Exclusion Criteria, Data Synthesis and Analysis

Defining the inclusion and exclusion criteria was an iterative process informed by published scoping review methodologies (37, 38). Initiatives reporting no involvement were excluded from further analysis. Initiatives were categorized as “no involvement” if the context of words such as “participation” were used to describe “research participants” (research subjects) only, rather than aligning with the concept of involvement already articulated (4). Reported impacts were excluded if they were phrased as anticipated future impacts (using terms such as “we expect”), rather than reporting real results. Initiatives reporting “data sharing” as the only type of involvement were also excluded. Initiatives reporting any other type of involvement, according to our definition, were included and proceeded to data extraction (structured categorization of extracted search data).

Extracted data was categorized (data synthesis) based on the following types of information; (**a**) the *method* of involvement (*how* people were involved) (24); (**b**) the *tasks* they were involved in (what people *did*) (24); (**c**) the *stage* of the research (using an expanded version of an existing framework (15), informed by INVOLVE) (39); (**d**) *who* was involved, for example “research participants,” “patients,” and “public” (informed by the Concannon “7Ps Framework” taxonomy) (16); (**e**) *reported facilitators or barriers* of involvement; and (**f**) publicly-reported *impacts* (informed by section 7 and 8 of the GRIPP2 framework) (16, 24).

As there is currently no standardized way to report and group methods of involving people or descriptions of people involved (24, 29), grouping was informed by methods of previous reviews [for example, grouping similar methods of involving people (24)] and by using previously established nomenclature (26, 33). The initial grouping (JN) was reviewed by other authors (PL). While previous reviews have used frameworks to label the “roles,” “degrees,” or “levels” of involvement or “control” (19, 24), we did not use these frameworks as they require subjective judgements to be made, often with insufficient data (26, 40–42).

## Results

Of the 96 initiatives searched, based on our criteria, only a third reported involving people in some capacity (32/96, 33%) (Table 1). These 32 initiatives were included in the final analysis (data synthesis).

**Table 1:** Summary of G44GH initiatives reporting public involvement.

**Name of Initiative/Organization**	**ID**	**Geographic Region (cohort size)**	**Reported methods of involving people**
100 k Wellness Project	1	North America (100,000)	Online tools, Other
Australian Genomics Health Alliance (AGHA)	8	Australia (1,800)	Formal groups, Other, Public events
Biobanking and Biomolecular resources Research Infrastructure (BBMRI)	11	Europe (N/A)	Formal discussion formats, Public events
Cancer MoonShot 2020	16	North America (20,000)	Generic involvement
Clinical Sequencing Exploratory Research (CSER)	21	North America (6,000)	Generic involvement
DECIPHER	24	International (21,475)	Formal groups
East London Genes and Health	26	Europe (100,000)	Formal groups, Generic involvement
Electronic Medical Records and Genomics (eMERGE)	27	North America (55,028)	Surveys
ELIXIR	28	Europe (N/A)	Consultation, Formal groups, Public events
France Genomic Medicine 2025	33	Europe (N/A)	Consultation, Generic involvement
Genome in a Bottle	35	International (N/A)	Generic involvement, Public events
Genomics England	37	Europe (100,000)	Consultation, Formal discussion formats, Formal groups, Generic involvement, Other, Public events, Surveys
H3Africa	41	Africa (60,000)	Formal discussion formats, Generic involvement
Implementing Genomics in Practice (IGNITE)	44	North America (73,000)	Formal groups, Public events
International Rare Diseases Research Consortium (IRDiRC)	50	International (N/A)	Formal groups, Generic involvement, Other, Public events
Kaiser Permanente Research Program on Genes, Environment, and Health (RPGEH)	52	North America (500,000)	Formal groups
Kaviar	53	North America (N/A)	Formal groups
Matchmaker Exchange	57	International (N/A)	Formal groups, Online tools
MSSNG	60	North America (10,000)	Formal groups
MyCode Community Health Initiative	62	North America (250,000)	Formal groups
MyGene2	63	International (500)	Online tools
openSNP	65	Europe (2,500)	Citizen science, Online tools, Surveys
Precision Medicine Initiative /“All of Us”	69	North America (10,00,000)	Citizen science, Formal groups, Formal discussion formats, Generic involvement, Online tools, Other, Public events, Surveys
Public Population Project in Genomics and Society (P3G)	72	International (N/A)	Formal groups, Online tools, Public events, Surveys
Qatar Genome Project	73	Asia (1,161)	Surveys
RD-Connect	74	Europe (2,500)	Formal discussion formats, Formal groups, Generic involvement, Newsletters, Online tools, Surveys
The Clinical Genome Resource (ClinGen)	84	North America (N/A)	Formal groups, Other
Tohoku Medical Megabank Project	86	Asia (150,000)	Formal discussion formats, Public events, Surveys
Transforming Genetic Medicine Initiative (TGMI)	88	Europe (N/A)	Public events
UK Biobank	92	Europe (500,000)	Consultation, Formal discussion formats, Formal groups, Generic involvement, Newsletters, Other, Public events, Surveys
Undiagnosed Diseases Network (UDN)	94	North America (8,000)	Formal groups
Vanderbilt's BioVU	96	North America (215,000)	Formal groups, Public events

*Initiatives from a database provided by the Global Alliance for Genomics and Health were searched for reports of public involvement (based on public domain websites). Each initiative has been assigned an ID number. The type (method) of involvement was categorized using specific criteria*.

### Reported Methods of Involvement

The reported methods of involving people were organized into categories, shown below in **bold**, with the number of total initiatives reporting each method shown in brackets:

- **Citizen science** (*n* = 2)**—**people involved beyond data collection, research design or data analysis, toward co-creation across all aspects of the scientific process (43);
- **Consultation** (*n* = 4)—an organized consultation or dialogue process;
- **Formal discussion** (*n* = 8)—formalized “focus groups,” forums or interview structures;
- **Formal groups** (*n* = 20)—a working group or committee (including ethics and data access committees, “scientific advisory groups” and “steering groups”);
- **Generic involvement** (*n* = 11)—informal, such as meetings, “partnership,” or an unspecified method;
- **Newsletters** (*n* = 2)**—**or mailing lists;
- **Online tools** (*n* = 7)—websites, social media, or online community hosting;
- **Public events** (*n* = 13)—with discussion—including initiatives hosting public debates, workshops, discussion spaces, or conferences;
- **Surveys** (*n* = 10)—including questionnaires; and
- **Other** (*n* = 7)—methods not described by other categories.

Some initiatives reported using multiple methods to involve people. Reports of involving people also showed that some methods, for example “formal discussion,” can use different modes of communication, including face to face, online (for example, “massive open online courses”), or a combination of the two.

**Figure 2:**
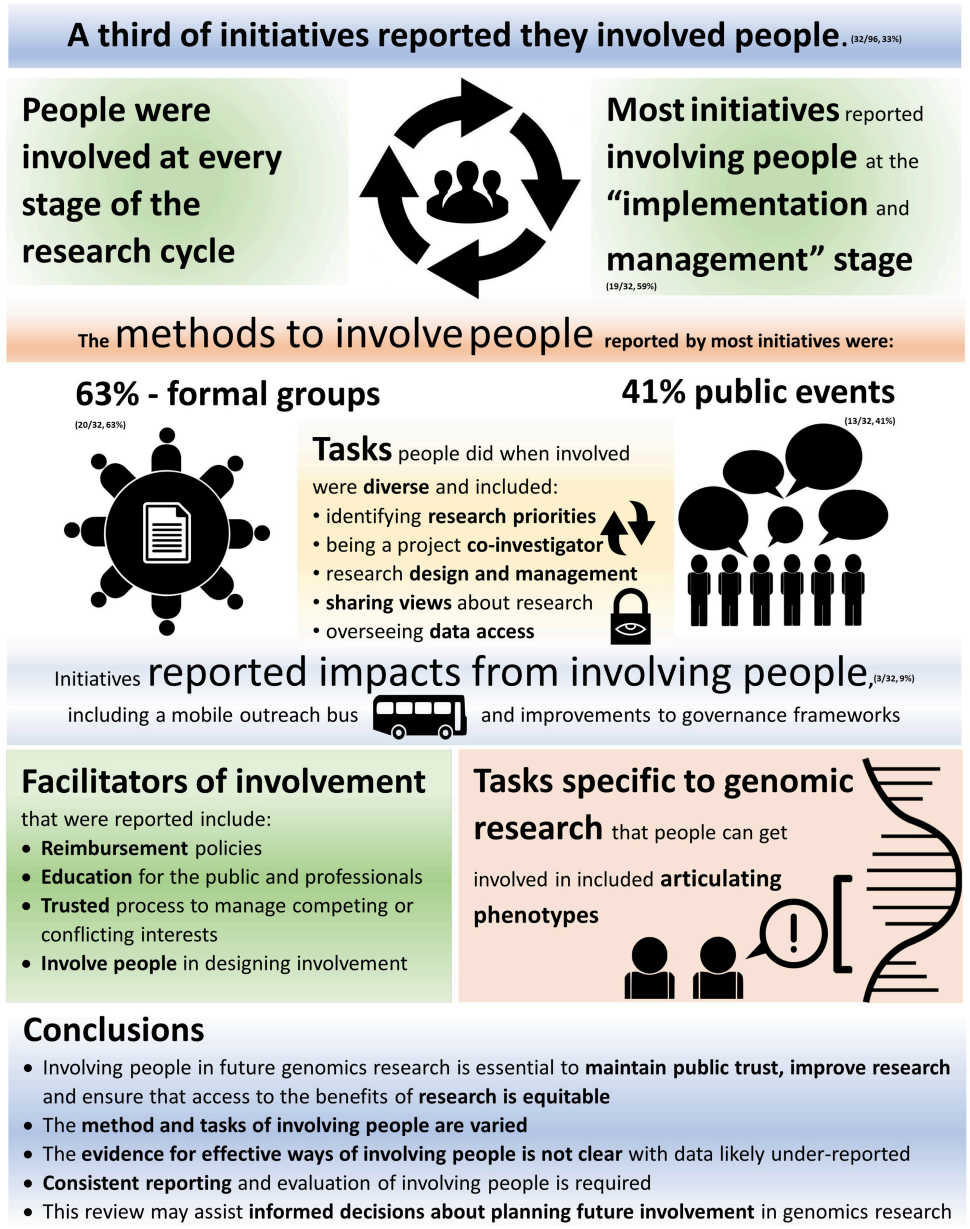
Summary of results.

Figure 2 summarizes overall findings from data synthesis. There was variability in the methods and tasks of involvement reported. This supports previous findings that involvement in biomedical research is diverse, varied, and described using different language (44).

### Reported Tasks of Involvement

The tasks people were involved in (what people did when they were involved) were diverse. Tasks included identifying research priorities related to people with specific diseases; communicating priorities to scientists, clinicians and health policy makers [IDs 11, 37, 50, 74]; designing or improving how people will be involved in the research [IDs 41, 50]; educating professionals involved in the research [ID 8]; developing workshops and conferences [IDs 44, 94]; offering culturally appropriate information about research to people in community groups [ID 37]; providing feedback on the cultural and linguistic appropriateness of public domain research documents [ID 96]; and translating information into “lay” language [ID 92]. Tasks also involved sharing views and perspectives about multiple aspects of research projects [ID 37, 92, 96]; articulating phenotypes [ID 65]; and being a project co-investigator [ID 21].

Some initiatives reported involving people in the task of giving feedback and sharing views and perspectives about the “acceptability” of specific aspects of the research design. For example, research management, governance [IDs 27, 41, 92, 72], accountability, planning, policy, protocols, data access, and data use [IDs 37, 74, 84, 92], consent, re-contact, withdrawal, confidentiality, benefit sharing, project closure, and recruitment [IDs 37, 62, 74, 92]. A number of initiatives also involved people in the task of sharing views and perspectives on issues of perceived social and ethical importance (including being told about potentially serious incidental findings) [IDs 37, 74, 96], or to scrutinize a project to ensure it aligned with public interest [ID 11].

While there are commonalities with the principles of involvement in other kinds of biomedical research, the review identified three novel tasks in relation to human genomics research not found in other reviews. These included involving people in phenotype articulation [ID 65], where people can describe the lived-experience of having specific genomic variations; articulating the variation in perspectives of people affected by different rare diseases [ID 74]; and collective governance, problem solving and improving code [ID 53; 65]. For example, Open SNP shared the code for the entire initiative using Github (a platform for sharing open-source code), inviting participants and other members of the public to scrutinize, contribute, and improve the code.

### Reported Stages of Involvement

Most reports of involvement were at the “implementation and management” stage of research (19/32, 59%), followed by “dissemination” (12/32, 38%), “evaluation,” and “study design” (both 9/32, 28%) and “data analysis” (8/32, 25%). The stage with the lowest number of initiatives reporting involvement was “funding” (1/32, 3%) with the next lowest being “identifying topics” and “prioritization” (both 4/32, 13%). Four initiatives reported involving people at every stage of research [IDs 21, 50, 69, 74].

### Reported Impacts of Involvement

Nearly 10% of the initiatives reporting involvement also reported impacts of the involvement (3/32, 9.4%). Three initiatives reported a total of eight distinct impacts as a direct result of involving people [IDs 37, 73, 92]. The method with the most reported impacts was “public events” (4/8, 50%), followed jointly by “formal discussion formats” and “surveys” (2/8, 25%). Actions taken as a result of involving people (impacts) included the creation of a mobile outreach bus [ID 37]; improvements to ethical and governance frameworks [ID 92] (45); and improved participant information and consent documents [ID 37] (46). Some impacts were reported as being a result of using a combination of methods.

### Reported Facilitators and Barriers to Involvement

A number of specific facilitators of involvement were reported, including: reimbursement policies [ID 21], with people involved paid for their time [IDs 92, 94], travel [IDs 74, 94], accommodation [ID 74] and expenses [IDs 74, 92]; education and learning opportunities for the general public [IDs 1, 11, 41]; ensuring people involved are informed and can make informed decisions [ID 11]; education for health professionals [IDs 41, 50]; providing opportunities to learn about how to involve people [IDs 41, 50]; and governance which is trusted by all stakeholders to be able to manage real or perceived competing or conflicting interests [ID 50]. The only barrier reported was limited venue size, which restricted the number of people who could be involved [ID 92]. This also implies a limited budget, which is an important but likely under-reported implicit limitation on all involvement methods.

## Discussion

This review provides an overview of reported public involvement occurring in prominent human genomics projects worldwide, during a period of rapid growth for genomics research. We identified significant variability in the way in which involvement occurs and is reported. The variation in reported involvement suggests diversity in both the ways people are being involved in genomics, and in the varied and emergent language used to report and describe involvement, consistent with other areas of biomedical research (8, 21). While there are similarities with the principles of involvement in other kinds of research, this review has identified three different tasks specific to genomics, not found in other reviews (44).

Because the results from this review suggest there is currently no standardized way of reporting involvement in human genomics, and therefore evaluating how people are involved, there is a risk that best-practice will be hard to define or even absent in future evidence reviews. This has implications, as the number of people involved in human genomic research is predicted to grow exponentially. Without a standardized framework to report and transparently evaluate ways people are involved, it will be difficult to create an evidence base to inform best-practice.

While a third of initiatives reported involvement, a majority of projects did not (64/96, 66%). Some prominent initiatives involving the genetic analysis of thousands of people did not refer to public involvement in any way. This is somewhat concerning given that involving the public has been identified as a crucial aspect of responsible research practice in genomics (1). Whilst we acknowledge the probable under-reporting of involvement activities on public-domain websites, we argue public involvement in human genomics research needs to increase.

Findings from this review also suggest it is best-practice to involve multiple stakeholders (including the public) in designing how people will be involved in research (co-design of the involvement plan), and to involve the public throughout the lifetime of a project in certain tasks (such as overseeing data access) and to evaluate the involvement with both qualitative and quantitative data.

Co-design of involvement strategies may improve how appropriate, effective, efficient and equitable they are. Seeking input from people into the design of planned methods of involvement by identifying what is considered “good practice” was reported by H3Africa [ID 41] and the International Rare Diseases Research Consortium (IRDiRC), and reported as a facilitator of involvement by the IRDiRC [ID 50]. The IRDiRC [ID 50] also reported both qualitative and quantitative data should be used to evaluate involvement, although there is currently no way to systematically collect and analyze such activity (47).

If involvement is more effective when the public are invited to help plan it, standardized reporting and evaluation will help make informed decisions at every stage of involvement from co-design through to evaluation.

### Implications for Policy and Practice

With the impact of some genomics research data likely to be measured in decades, some of the initiatives offer a useful insight into planning and funding of sustainable (long-term) involvement for the entirety of an initiative's lifetime (9). For example, Genomics England [ID 37] and the UK Biobank [ID92], as exemplars, both reported multiple ways of involving people, at different stages of the research cycle, conducted over a number of years. Other initiatives, such as the International Rare Diseases Research Consortium (IRDiRC) [ID 50] and the Public Population Project in Genomics and Society (P3G) [ID 72], also publicly stated the importance of planning sustainable involvement over the duration of a project. These initiatives demonstrate a standard of involving people which could eventually be used to inform international best practice.

The IRDiRC also reported that involving people throughout an entire project helped maintain trust by scrutinizing and managing competing or conflicting interests [ID 50]. Similarly, the UK Biobank reported that involving people in ethics and governance should not be one-off and must be ongoing [ID 92]. The method of using “formal groups” was more common for more complex or ongoing tasks such as overseeing data access, policy development, research management and improving research protocols.

Some initiatives, such as openSNP, reported tasks that were specific to genomics research, such as articulating phenotypes [ID 65]. Involvement in this kind of task might have important implications when working to usefully describe people's subjective lived-experiences across multiple languages, for example, rare diseases and mental health (48).

Public involvement in articulating phenotypes also suggests that the traditional boundaries between terms such as “research,” “healthcare,” “patients,” “research participants,” and “the public” may be increasingly challenged by the methodology of future genomic research (49). Findings from this review show that both “the public” and “patients” are already involved in every stage of research, including collecting and analyzing data (49). Any future standardized reporting of involvement will need to keep pace with the continually evolving language to describe not only what research is, but who is involved and how.

### Limitations

While the database hosted by GA4GH includes many of the most prominent human genomics research initiatives worldwide, the database is not exhaustive. There are several known genomics initiatives which involved people that were not part of the database. Therefore, the GA4GH selection cannot be considered systematic or representative. However, it does provide a reasonable indication and snapshot of the current global landscape in human genomics research up to November 2017. After the review was completed, GA4GH shared a new a database with 220 initiatives (50), presenting an opportunity for future reviews. The addition of so many new projects to the database reflects the rapid pace of growth in human genomics research.

Our data collection was limited to self-reported information reported on English language websites only. This likely under-reports the total amount of public involvement occurring. For example, some initiatives may have conducted involvement, and not reported it publicly. This indicates a current lack of standardization or best-practice in reporting involvement activities in human genomics research, which we feel could be improved.

Of the public involvement activities reported, we did not systematically follow up reports to confirm they had taken place, or if involvement was “consequential” (27–29). While this is a limitation of the review, it also reflects the inconsistent and often incomplete ways genomics research initiatives report impacts of involving people. For example, the impact of how involvement influenced research was only reported by three projects—Genomics England [ID 37], the Qatar Genome Project [ID 73] and the UK Biobank [ID 92].

A number of reported methods did not provide sufficient information to make a clear decision about how to group a method. For example, many reports of involvement simply referred to a “workshop,” “meeting,” or other “public events,” where people were able to get involved by sharing views and perspectives. As a result there is potentially significant overlap between some methods, which could have been articulated more clearly if more data were available. Similarly, while detailed data was extracted about “who” was involved, ways of grouping terms such as “research participants,” “patients,” and “public” requires further development to co-create standardized definitions.

The systematic searching of domains with the Google site search function relies on Google servers having carried out a “website crawl,” where data from the website is indexed (51). As the search and indexing process is partially opaque (not open-source), this method cannot be considered “exhaustive.” However, it is an appropriate supplementary search strategy for this scoping review.

Reports of “data sharing” were excluded, as they were not considered as public involvement. While sharing data may enable people to be involved in some tasks (for example, in analyzing data), data sharing is not necessarily an indicator that people were involved in the analysis of data. The complexity within the term “data sharing” in genomics, and how people can be involved in the analysis and interpretation of data, also requires further consideration (52–54).

## Conclusion

Involving people in the future of genomics research is an essential aspect in maintaining public trust, improving research outcomes, and ensuring that access to the benefits of genomics research is equitable (1, 14, 49). The limited number of initiatives reporting public involvement and its impact in this study suggests there would be significant value in developing a more systematic method of both reporting and evaluating how people are involved in human genomics research. Data from such reporting could provide the evidence required to inform future policy around involvement of the public, as human genomics research continues to grow.

## Author Contributions

All authors listed have made a substantial, direct and intellectual contribution to the work, and approved it for publication.

### Conflict of Interest Statement

The authors declare that the research was conducted in the absence of any commercial or financial relationships that could be construed as a potential conflict of interest.

## Supplementary Material

Table S1 GA4GH database and results table. This table combines a database of human genomics research projects hosted by the Global Alliance for Genomics and Health with a summary of results from a scoping review of currently reported public involvement reported on public domain websites. The data is organized alphabetically, with organizations reporting involvement listed first.

Systematic search method This document describes the search method, including how standard and adaptive TERMS were used to search the public domain websites of all the included initiatives in the GA4GH database for reports of involvement and any impacts.
