# Supplementary material for: Public Involvement in Global Genomics Research: A Scoping Review
Source: Front Public Health. 2019 Apr 9;7:79. doi: 10.3389/fpubh.2019.00079 (PMC6467093; doi:10.3389/fpubh.2019.00079)
Supplement: Systematic search method — This document describes the search method, including how standard and adaptive TERMS were used to search the public domain websites of all the included initiatives in the GA4GH database for reports of involvement and any impacts. [file Table_2.docx]

Systematically searching sites for ‘public involvement’ and related concepts

Using the search strategy described below, the public domain websites of all the included initiatives in the GA4GH database were searched for reports of involvement and any impacts. This document describes Stage 1 and Stage 2 of the scoping review in more detail. The graphic below visually represents the different phases at each stage of the review.

# Stage 1 – Defining “involvement” and the search strategy

We conducted a narrative review of systematic reviews to inform our search terms. In addition we used similar studies and existing frameworks to inform the word list ^1–14^. We developed a criteria to define ‘involvement’ based on the International Association for Public Participation’s participation spectrum and other studies^9,10,15,16^. The final list was checked by the research team after being tested. Below are the search terms included in the main search.

## Main search terms to describe ‘involvement’

An asterisk (*) denotes that all possible grammatical variations of the nouns used to describe people involved.

- Involvement (involv*)
- Engagement (engag*)
- Partnering (partner*)

## Main search terms to describe people involved

An asterisk (*) denotes that all possible grammatical variations of the nouns used to describe people involved.

- public*
- communit*
- consumer*
- patient*
- stakeholder
- user*
- citizen*
- Lay (‘people’ included to exclude phrases such as ‘involved laying down’)
- Patient
- PPI (an acronym commonly used in the UK which stands for ‘patient and public involvement’)

# Stage 2 – Searching websites (data extraction)

Public domain websites of all the initiatives in the GA4GH database were searched for reports of involvement and associated impacts.

## **Phase one: manual search**

Public domain websites of all initiatives in the GA4GH database were manually searched for reports of involvement and associated impacts.

## Phase two: Adaptive search terms added

During the manual search, adaptive (context dependent) search terms are added to phrase generation table. The adaptive search terms which were added to the phrase generation table were:

- lay*
- carer*
- volunteer*
- population* ^1^
- group*
- residents (geographical grouping) ^2^
- participa* (context dependant)
- representative
- payer*
- taxpayer*
- customer* ^7^
- client* ^7^
- advocate*
- civil societ*

When any of these terms returned a result that was within the inclusion criteria, the exact search string was recorded.

## Phase three: systematic site search

### Phrase-generation table

Commercial search engines like Google do not allow an asterisk (*) to denote all variations in grammatical endings, so searching for all possible variations must be done manually. Search engines like Google do allow the ‘OR’ operator (up to 35 words per search), meaning multiple variations can be searched at once. Once the list of words was finalised, the variations were generated in order to systematically create search strings. This checklist was used to ensure all possible verb and noun forms variations were used.

**Verb forms**

- Base (root) form or infinitive (involve, engage, partner)
- Active or plural form (involves, engages, partners), used as the present indicative in the third-person singular
- Past tense (involved, engaged, partnered)
- present participle, gerund, verbal and deverbal nouns (involving, engaging, partnering)

**Nouns**

- Singular (involvement, engagement, partnership)
- Plural (involvements*, engagements*, partnerships)

*Not found in prototype searches

The table below demonstrates this process for systematically combining the various words to describe people involved with the terms used to describe involvement.

| **Term for ‘the public’** | **Involvement** | **Engagement** | **Partnering** |
| --- | --- | --- | --- |
| public* | “public involvement” OR “public involved” OR “involving public” OR “involves public” OR “involving the public” | “public engagement” OR “public engaged” OR “engaged public” OR “engaging the public” | “public partner” OR “public partners” OR “public partnership” OR “public partnerships” OR “public partnering” OR “partnering with the public” |
| communit* | “community involvement” OR “communities involved” OR “involving communities” OR “involved communities” OR “involve communities” OR “involves the community” | “community engagement” OR “community engaged” OR “engaged community” OR “community engages” OR “engaged community” OR “engaging the community” OR “community engaging” | “community partner” OR “community partners” OR “community partnership” OR “community partnerships” OR “community partnering”  OR “community partnered” OR “partnering with the community” |
| consumer* | “consumer involvement” OR “consumers involved” OR “involving consumers” OR “involved consumers” OR “involve consumers” OR “involves consumers” | “consumer engagement” OR “consumers engaged” OR “engaged consumer” OR “engaged consumers” OR “consumer engages” OR “engaged consumer” OR “engaging the consumer” OR “engaging the consumers” OR “consumer engaging” | “consumer partner” OR “consumer partners” OR “consumer partnership” OR “consumer partnerships” OR “consumer partnering”  OR “consumers partnered” OR “partnering with the consumers” |
| patient | “patient involvement” OR “patients involved” OR “involving patients” OR “involved patients” OR “involve patients” OR “involves patients” | “patient engagement” OR “patients engaged” OR “engaged patient” OR “engaged patients” OR “patient engages” OR “engaged patient” OR “engaging the patient” OR “engaging the patients” OR “patients engaging” | “patient partner” OR “patient partners” OR “patient partnership” OR “patient partnerships” OR “patient partnering”  OR “patients partnered” OR “partnering with the patients” |
| stakeholder | “stakeholder involvement” OR “stakeholder involved” OR “involving stakeholders” OR “involved stakeholders” OR “involve stakeholders” OR “involves stakeholders” | “stakeholder engagement” OR “stakeholders engaged” OR “engaged stakeholder” OR “engaged stakeholders” OR “stakeholders engage” OR “stakeholder engages” OR “engaged stakeholder” OR “engaging the stakeholder” OR “engaging the stakeholder” OR “stakeholders engaging” | “stakeholder partner” OR “stakeholder partners” OR “stakeholder partnership” OR “stakeholder partnerships” OR “stakeholder partnering”  OR “stakeholder partnered” OR “partnering with the stakeholders” |
| user* | “user involvement” OR “user involved” OR “involving users” OR “involved users” OR “involve users” OR “involves users” | “user engagement” OR “user engaged” OR “engaged user” OR “engaged users” OR “users engage” OR “user engages” OR “engaged users” OR “engaging the users” OR “engaging the user” OR “user engaging” | “user partner” OR “user partners” OR “user partnership” OR “user partnerships” OR “user partnering”  OR “user partnered” OR “partnering with the users” |
| citizen* | “citizen involvement” OR “citizen involved” OR “involving citizens” OR “involved citizens” OR “involve citizens” OR “involves citizens” | “citizen engagement” OR “citizen engaged” OR “engaged citizen” OR “engaged citizens” OR “citizens engage” OR “citizen engages” OR “engaged citizens” OR “engaging the citizens” OR “engaging the citizen” OR “citizen engaging | “citizen partner” OR “citizen partners” OR “citizen partnership” OR “citizen partnerships” OR “citizen partnering”  OR “citizen partnered” OR “partnering with the citizens” |
| Lay (‘people’ included to exlude phrases such as ‘involved laying down’) | “lay involvement” OR “lay involved” OR “involving lay people” OR “involved lay people” OR “involve lay people” OR “involves lay people” | “lay engagement” OR “lay engaged” OR “engaged lay” OR “engaged lay” OR “lay engage” OR “lay engages” OR “engaged lay” OR “engaging the lay” OR “engaging the lay” OR “lay engaging” | “lay partner” OR “lay partners” OR “lay partnership” OR “lay partnerships” OR “lay partnering”  OR “lay partnered” OR “partnering with lay” |
| Public and/& patient  Patient and& Public  “PPI involvement” included as it is often misused | “public and patient involvement” OR “patient and public involvement” or “involving patients and the public” OR “involving the public and patients” OR “ppi involvement” | “public and patient engagement” OR “patient and public engagement” or “engaging patients and the public” OR “engaging the public and patients” OR “ppi engagement” | “public and patient partnerships” OR “patient and public partnerships” or “partnering with patients and the public” OR “partnering with the public and patients” OR “ppi partnerships” OR “ppi partnering” |

The words ‘dialogue’, ‘consultation’ and variations of ‘co-production’ are sometimes used to describe involving people, so these terms were also searched for.

| **Dialogue** | **Consult** | **co-** |
| --- | --- | --- |
| “public dialogue” OR “public dialogues” OR “dialogue with the public” | “public consultation” | “co-production” OR “co-produced” OR “co-created” OR “co-designed” OR “co-design” |

### Systematic site search

Once the manual search was completed, websites were searched using the ‘site search’ function on the Google search engine. This method relies on Google servers having carried out a ‘website crawl’, where data from the website is indexed ^17^. While this method cannot be called ‘exhaustive’, it is an appropriate sampling technique for this scoping review. In addition, the limitations of the Google ‘site search’ function (with regards to Boolean operators and a maximum character limit of 35 characters per search) were partially overcome by the ‘phrase generation table’ which was used by authors to manually create an exhaustive list of search operators.

Once the search string was generated in the phrase generation table it was entered into the Google search engine. For example, this search string returned 4 results:

*site:www.ukbiobank.ac.uk/* “public involvement” OR “public involved” OR “involving public” OR “involves public” OR “involving the public”

If a term such as ‘patient’ was commonly used on a site (and thus had more than 10 pages of search results) ‘intext’ operators were used to refine the search to only return page results where another specific term appears. For example:

*site:www.irdirc.org patient intext:participation*

When search strings returned a result, these were logged in the extraction document.

# References

1. Nilsen ESE, Myrhaug HTH, Johansen M, Oliver SR, Oxman ADA. Methods of consumer involvement in developing healthcare policy and research, clinical practice guidelines and patient information material. In: Nilsen ES, ed. *Cochrane Database of Systematic Reviews*. Chichester, UK: John Wiley & Sons, Ltd; 2006:CD004563. doi:10.1002/14651858.CD004563.pub2.

2. Collins M. PiiAF The Public Involvement Impact Assessment Framework Guidance. http://piiaf.org.uk/documents/piiaf-guidance-jan14.pdf. Published 2014. Accessed October 4, 2017.

3. Brett J, Staniszewska S, Mockford C, et al. A Systematic Review of the Impact of Patient and Public Involvement on Service Users, Researchers and Communities. *Patient*. 2014;7(4):387-395. doi:10.1007/s40271-014-0065-0.

4. Shippee ND, Domecq Garces JP, Prutsky Lopez GJ, et al. Patient and service user engagement in research: A systematic review and synthesized framework. *Heal Expect*. 2015;18(5):1151-1166. doi:10.1111/hex.12090.

5. Brett J, Staniszewska S, Mockford C, et al. Mapping the impact of patient and public involvement on health and social care research: a systematic review. *Health Expect*. 2014;17(5):637-650. doi:10.1111/j.1369-7625.2012.00795.x.

6. Avard D, Jean MS, Grégoire G, Page M. Public involvement in health genomics: The reality behind the policies. *Int J Consum Stud*. 2010;34(5):508-524. doi:10.1111/j.1470-6431.2010.00914.x.

7. Mitton C, Smith N, Peacock S, Evoy B, Abelson J. Public participation in health care priority setting: A scoping review. *Health Policy (New York)*. 2009;91(3):219-228. doi:10.1016/j.healthpol.2009.01.005.

8. Lander J, Hainz T, Hirschberg I, Strech D. Current practice of public involvement activities in biomedical research and innovation: A systematic qualitative review. *PLoS One*. 2014;9(12):e113274. doi:10.1371/journal.pone.0113274.

9. Kelty C, Panofsky A. Disentangling public participation in science and biomedicine. *Genome Med*. 2014;6(1):8. doi:10.1186/gm525.

10. Rogers M, Bethel A, Boddy K. Development and testing of a medline search filter for identifying patient and public involvement in health research. *Health Info Libr J*. 2017;34(2):125-133. doi:10.1111/hir.12157.

11. Woolley JP, McGowan ML, Teare HJA, et al. Citizen science or scientific citizenship? Disentangling the uses of public engagement rhetoric in national research initiatives. *BMC Med Ethics*. 2016;17(1):33. doi:10.1186/s12910-016-0117-1.

12. Staley K. *Exploring Impact: Public Involvement in NHS, Public Health and Social Care Research*. National Institute for Health Research; 2009. http://www.invo.org.uk/posttypepublication/exploring-impact-public-involvement-in-nhs-public-health-and-social-care-research/. Accessed March 17, 2017.

13. Knoppers BM, Leroux T, Doucet H, et al. Framing genomics, public health research and policy: points to consider. *Public Health Genomics*. 2010;13(4):224-234. doi:https://dx.doi.org/10.1159/000279624.

14. Domecq JP, Prutsky G, Elraiyah T, et al. Patient engagement in research: a systematic review. *BMC Health Serv Res*. 2014;14(1):89. doi:10.1186/1472-6963-14-89.

15. National Institute for Health Research. Patient and public involvement in health and social care research. 2014. https://www.nihr.ac.uk/about-us/CCF/funding/how-we-can-help-you/RDS-PPI-Handbook-2014-v8-FINAL.pdf. Accessed February 2, 2018.

16. Concannon TW, Meissner P, Grunbaum JA, et al. A new taxonomy for stakeholder engagement in patient-centered outcomes research. *J Gen Intern Med*. 2012;27(8):985-991. doi:10.1007/s11606-012-2037-1.

17. Google. How Google’s Site Crawlers Index Your Site. https://www.google.com/search/howsearchworks/crawling-indexing/. Accessed February 26, 2019.
